# Aquaporin-4 and Cerebrovascular Diseases

**DOI:** 10.3390/ijms17081249

**Published:** 2016-08-11

**Authors:** Heling Chu, Chuyi Huang, Hongyan Ding, Jing Dong, Zidan Gao, Xiaobo Yang, Yuping Tang, Qiang Dong

**Affiliations:** 1 Department of Neurology, Huashan Hospital, State Key Laboratory of Medical Neurobiology, Fudan University, No. 12 Mid. Wulumuqi Road, Shanghai 200040, China; lindadoctor7455@gmail.com (H.C.); 0422012@fudan.edu.cn (H.D.); 16211220018@fudan.edu.cn (J.D.); 0456184@fudan.edu.cn (Z.G.); 0531015@fudan.edu.cn (X.Y.); 2 Department of Neurology, Shanghai Jiaotong University Affiliated Sixth People’s Hospital, No. 600 Yishan Road, Shanghai 200030, China; huang-zy13@mails.tsinghua.edu.cn; 3 Department of Neurology, Jinshan Hospital, Fudan University, No. 1508 Longhang Road, Shanghai 201508, China

**Keywords:** aquaporin-4, cerebral ischemia, intracerebral hemorrhage, subarachnoid hemorrhage

## Abstract

Cerebrovascular diseases are conditions caused by problems with brain vasculature, which have a high morbidity and mortality. Aquaporin-4 (AQP4) is the most abundant water channel in the brain and crucial for the formation and resolution of brain edema. Considering brain edema is an important pathophysiological change after stoke, AQP4 is destined to have close relation with cerebrovascular diseases. However, this relation is not limited to brain edema due to other biological effects elicited by AQP4. Till now, multiple studies have investigated roles of AQP4 in cerebrovascular diseases. This review focuses on expression of AQP4 and the effects of AQP4 on brain edema and neural cells injuries in cerebrovascular diseases including cerebral ischemia, intracerebral hemorrhage and subarachnoid hemorrhage. In the current review, we pay more attention to the studies of recent years directly from cerebrovascular diseases animal models or patients, especially those using AQP4 gene knockout mice. This review also elucidates the potential of AQP4as an excellent therapeutic target.

## 1. Introduction

Aquaporins (AQPs) are a family of water channel proteins and famous for water transportation under physical and pathological conditions. Since the first water channel, termed AQP1, was discovered by Peter Agre in 1992 [1], at least 13 AQP members have been found in mammals [2]. A consensus motif is a common characteristic of all members of AQPs, which is essential for pore formation [3]. Apart from the pure water channel (AQP0, -1, -2, -4, -5, -6, and -8), there are a subset of AQPs that also transport glycerol called aquaglyceroporins (AQP3, -7, -9, and -10) [4].

In 1994, Agre’s group isolated the fourth mammalian member of the aquaporin water channel family (AQP4) by homology cloning, which regulated body water balance and mediated water flow within the central nervous system (CNS) as the osmoreceptor [5]. AQP4 is the most abundant water channel in CNS and predominantly expressed in astrocyte foot processes surrounding capillaries astrocyte processes which are comprised of the glial limiting membrane and in ependymal cells [6,7]. It is crucial for the formation and resolution of brain edema. Besides CNS water balance maintenance, by means of AQP4 knockout animals, several other biological effects of AQP4 have been demonstrated, including neural signal transduction regulation, synaptic plasticity, astrocyte migration, neurogenesis and neuroinflammation [8,9,10,11,12].

Cerebrovascular diseases are conditions caused by problems with brain vasculature, which mainly contain ischemic and hemorrhagic stroke. These diseases have a high morbidity and mortality throughout the world. Because brain edema is an important pathophysiological change after stoke, AQP4 is destined to have close relation with cerebrovascular diseases. However, this relation is not limited to brain edema. This review briefly introduces the structure and function of AQP4 and focuses on the effects of AQP4 on stroke.

## 2. Structure and Function of AQP4

### 2.1. Structure and Distribution of AQP4

**Figure 1:**
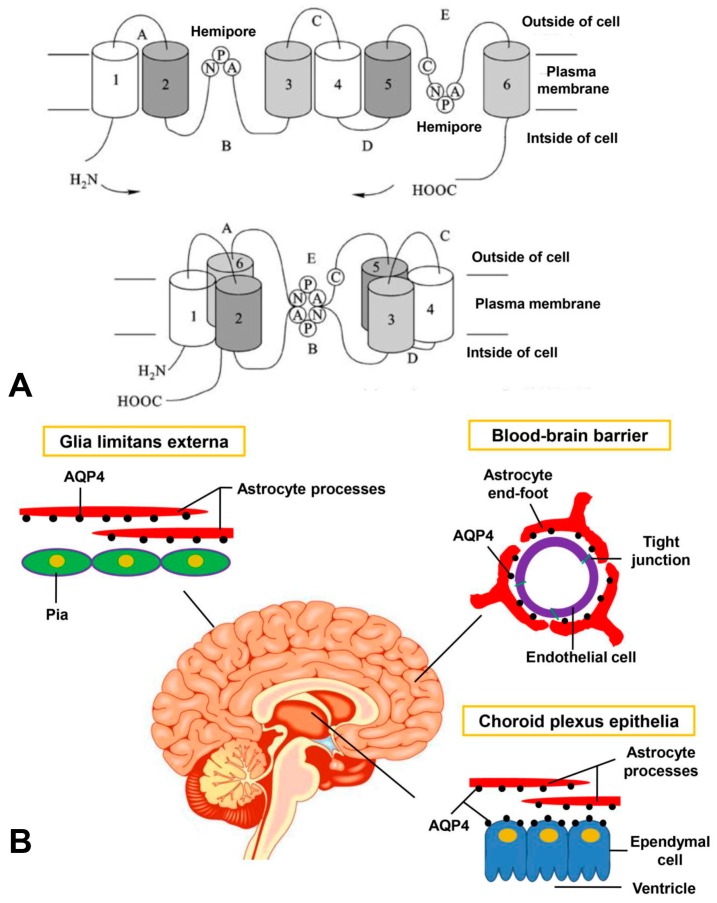
The structure and distribution of aquaporin-4 (AQP4). (**A**) AQP4 has six transmembrane domains (1–6) and five connecting loops (A–E). Loops B and E contain highly conserved “NPA” motifs (hemipores) that overlap midway creating a highly selective water pore; (**B**) AQP4 is polarized at the astrocyte processes facing cerebrospinal fluid (CSF)–brain and blood–brain barrier. Ependymal cells have basolateral expression of AQP4 [13,14,17].

AQP4 monomers consist of six helical, membrane-spanning domains and two highly conserved Asn-Pro-Ala (NPA) motifs that create a narrow aqueous pathway [13] (Figure 1A). Similar to other aquaporins, AQP4 monomers also assemble as tetramers. Importantly, AQP4 tetramers further cluster in the plasma membrane forming crystal-like supramo-lecular assemblies, termed orthogonal arrays of particles (OAPs). OAPs can be visualized in membranes by freeze-fracture electron microscopy whichare originally confirmed to be formed by AQP4 in AQP4-transfected Chinese hamster ovary cells [14,15]. AQP4 has two major isoforms: M1 and M23, which are transcribed from two different initiation sites on the same gene. M1 is a relatively long isoform with translation initiation at Met-1, while M23 is a shorter one with translation initiation at Met-23 [16].

Although AQP4 is the most abundant water channel in the brain, it is only detected in the plasma membrane of astrocytes and ependymal membranes since its discovery over two decades. Its location can be characterized as the cell surfaces of the blood–brain barrier (BBB) and cerebrospinal fluid (CSF)–brain barrier. Therefore, AQP4 is expressed in astrocyte foot processes surrounding capillaries, astrocyte processes which are comprised of the glial limiting membrane, ependymal cells and subependymal astrocytes [7,18] (Figure 1B). Besides, it was also found that AQP4 mRNA and protein are expressed by reactive microglial cells. However, this is still controversial because of a lack of support from further study [19,20]. The polarized distribution of AQP4 depends on some proteins also with polarized expressionin astrocytes. α-syntrophin, a member of the dystrophin associated protein complex, plays an important role in anchoring of AQP4 to astrocyte end-foot processes [21,22]. Besides, the matrix constituent agrin is also responsible for AQP4 polarization [21,23].

### 2.2. Animal Models for Studying Function of AQP4

Currently, no effective and specific AQP4 inhibitors have been developed. AQP4 knockout mice play essential roles in exploring AQP4 function. Since all properties are similar to wild type mice except absence of AQP4, AQP4 knockout mice are excellent candidates for AQP4 study. Numerous reports have revealeddifferentAQP4 functions through comparing AQP4 deletion mice with wild type mice [8,9,10,11,12]. There are mainly three research groups that have reported AQP4 knockout lines. Verkman’s group, from San Francisco, USA, first generated AQP4 knockout lines in 1997, though they revealed many biological properties of AQP4 [24]. Afterwards, Hu’s group from Nanjing, China and the group of Ottersen and Nagelhus from Oslo, Norway also generated AQP4 knockout lines [25,26]. However, there are still some differences in the properties among the AQP4 null mice lines. For example, brain morphology and BBB integrity are not affected in AQP4 deletion mice from San Francisco and Oslo [20,26], which are distinct from those of Nanjing [27], suggesting tiny differences may be produced during the generating process.

Besides AQP4 deletion, there is an alternative tool model; that is, mice lacking polarized AQP4 expression. It has been proven that α-syntrophin deficient mice lack polarized expression of AQP4, which are the most commonly used model with depolarized expression of AQP4 [21]. This model demonstrates the significance of polarized distribution of AQP4 in its biological properties. In α-syntrophin-null mice, development of brain edema of an experimental acute hyponatremia model was delayed and K^+^ clearance in epileptic seizures was prolonged, which is in accordance with AQP4 deletion mice [28,29]. Therefore, the AQP4 depolarized expression model can be considered as an alternative approach to test AQP4 function.

### 2.3. AQP4 and Brain Edema

Brain edema is excess accumulation of fluid in the intracellular or extracellular spaces of the brain. Brian edema can result from several brain pathologies, including brain trauma, cerebrovascular diseases, brain tumors, brain inflammation and metabolic diseases [30]. Brain edema may aggravate the primary diseases. There are four types of brain edema: cytotoxic, vasogenic, osmotic and interstitial edema, with the former two being the most common and classic [31]. Cytotoxic edema is intracellular accumulation of water owing to energy failure resulting from impairment of the sodium and potassium pump in cell membrane. Astrocytes are the major cell type involved in cytotoxic edema [32]. The typical cytotoxic edema can be seen in early ischemia or hypoxia, cerebral malaria and hyponatremia. Vasogenic edema occurs due to disruption of BBB, which results in entry of intravascular proteins and fluid into extracellular space [33]. This kind of brain edema is usually found in brain tumors, focal inflammation, abscess and late ischemia. It has been revealed the existence of a brain-wide paravascular pathway for CSF and interstitial fluid exchange termed “glymphatic” system [34], which contributes greatly to the two types of brain edema. Polarized distribution of AQP4 plays an important role in this pathway [35]. By means of observation to appropriate brain edema models in AQP4 null mice versus wild types, it has been proven that AQP4 plays an important role in regulation of the two types of edema. Manley and colleagues established two models of cytotoxic edema, acute water intoxication and early cerebral ischemia, and demonstrated brain edema was significantly reduced in AQP4-deficient mice [36]. Besides, it was reported that AQP4-deficient mice had remarkably lower brain water accumulation in acute bacterial meningitis, another cytotoxic edema model [37]. In addition, mice lacking polarized AQP4 expression also had a reduction of water influx after early cerebral ischemia [28]. As to vasogenic edema, Papadopoulos et al. illustrated AQP4 knockout mice had elevated brain water in three vasogenic edema models: intracerebral fluid infusion, focal cortical freeze injury and brain tumor implantation [38]. Therefore, it can be concluded that AQP4 is involved in the formation of cytotoxic edema and elimination of vasogenic edema. Whereas, the brain edema formed by many CNS diseases is usually not a single type, and there are also some changes in brain edema types during the course. Therefore, the roles of AQP4 in those diseases are also complicated, and appropriate animal models are required for continuous and dynamic observation.

### 2.4. Other Function of AOP4

Besides regulation of brain edema, AQP4 can elicit multiple biological effects. Here, we briefly describe the important ones. Because AQP4 is selectively expressed in astrocytes, it is essential for astrocytes function. Verkman’s group demonstrated that cultured astrocytes derived from AQP4 null mice had markedly low migration efficiency towards the wound [10]. Moreover, it was verified using in vivo experiments, and it was found that astrocytes of wild type mice migrated faster than AQP4 knockout mice [39]. It was indicated that AQP4 promoted migration of astrocytes, which was beneficial for formation of glial scar. Meanwhile, AQP4 also modulates brain excitability in epilepsy [40]. Binder et al. demonstrated that the latency to generalized seizures was significantly lower in wild-type mice comparing with AQP4 deletion mice [41]. This group then explored the mechanism and the results showed AQP4 knockout mice increased seizure duration via slowing potassium kinetics [42]. α-syntrophin-null mice exhibited similar effects of prolonging potassium ions clearance [28]. However, the role of AQP4 in potassium ions buffering is controversial as Haj-Yasein et al. reported that AQP4 removal did not affect potassium ions recovery following synaptic activation [43]. Besides potassium ions, it was also reported AQP4 null mice had a reduction of astrocytic calcium ions spikes initiated by hypoosmotic stress, suggesting AQP4 is associated with calcium signal transduction [8]. Moreover, AQP4 has a potential to influence synaptic plasticity [44]. Skucas and colleagues demonstrated that absence of AQP4 selectively impaired neurotrophin-dependent synaptic plasticity [9]. Consistently, research from other groups also shows AQP4 is crucial for maintaining normal consolidation of long-term hippocampus-dependent memories by promoting incorporation of new neurons into spatial memory networks [45]. In addition, AQP4 is considered to promote neurogenesis. Hu’s group showed AQP4 deletion inhibited the proliferation, survival, migration and neuronal differentiation of neural stem cells derived from the subventricular zone by disrupting intracellular calcium ions dynamics [46]. Their following experiment using a depression model indicates AQP4 is required for the antidepressive action of fluoxetine through regulating adult hippocampal neurogenesis [11].

## 3. Cerebral Ischemia

### 3.1. Expression of AQP4

The pathogenic process and pathophysiological mechanism of the corresponding clinical diseases are properly simulated by the current brain ischemia models. There are mainly focal ischemic models: middle cerebral arterial occlusion (MCAO), including permanent and transient MCAO and global ischemic models: two or four-vessel occlusion in animal experiments. The well-known cell culture model is oxygen and glucose deprivation (OGD) with or without reoxygenation. Studies on AQP4 expression after cerebral ischemia mainly focus on animal or cell models. It is well-known that AQP4 mRNA and protein are up-regulated at 30 min after permanent MCAO [47]. After transient MCAO of rat pup, AQP4 expression was increased on astrocytic end-feet in the border regions of injured tissues at 24 h, lasting at least 72 h and normalized at 28 days, which was in accord with brain edema showed by Magnetic resonance imaging (MRI) [48]. A continuous and dynamic observation was carried out in another research using a MCAO and reperfusion model, and the results demonstrated AQP4 expression was significantly increased on astrocyte end-feet both in the core and in the border of the lesion with two peaks: 1 h and 48 h [49]. However, a recent study of a global cerebral ischemic model showed no marked change of AQP4 within 48 h [50]. In astrocyte culture, Chikako et al. found expression of AQP4 was significantly decreased by OGD injury, but gradually recovered after reoxygenation with a significant up-regulation after 16 h [51]. Other research also showed up-regulation of AQP4 24 h after OGD/reoxygenation [52]. There are also studies from cerebral infarction patients. It was demonstrated AQP4 was only expressed on astrocytes and was highly localized on their end feet facing the outer surface of capillaries [53,54]. Stokum and coworkers’ work focused on AQP4 expression in different cerebral zones of both infarction patients and animal models. Their results revealed in cortex perivascular AQP4 was reduced with an unchanged AQP4 protein abundance, while an increase of perivascular and plasmalemmal AQP4 was observed in white matter with a 2.2- to 6.2-fold increase in AQP4 isoform abundance. Meanwhile, ischemic white matter swelled by approximately 40%, while cortex swelled by approximately 9% [55]. Accordingly, it can be concluded that AQP4 expression after cerebral ischemia tends to be up-regulated, although the specific change forms may be different due to different models.

### 3.2. Regulation of AQP4 after Cerebral Ischemia

AQP4 expression is regulated by some signal transduction pathways in some in vivo and in vitro experiments. It was reported that AQP4 was down-regulated by activating protein kinase C (PKC) pathway by hydrogen sulfide or melatonin after MCAO, suggesting PKC pathway is essential for AQP4 down-regulation [56,57]. Mitogen-activated protein kinase (MAPK) pathways include three main members: extracellular signal-regulated kinase (ERK), C-Jun amino-terminal kinase (JNK) and p38-MAPK. There are two studies using astrocyte OGD/reoxygenation model, but the results are a little different. AQP4 was up-regulated by activation of ERK and p38-MAPK pathways [52], while JNK and p38-MAPK pathways were positive in another study [51]. The distinction may be owing to different experimental parameters (OGD 4 h vs. OGD 6 h) and the fact that cross talk exists generally among MAPK family. Therefore, MAPK pathways mainly have an important role in AQP4 up-regulation.

### 3.3. Effects of AQP4 on Ischemic Edema

**Figure 2:**
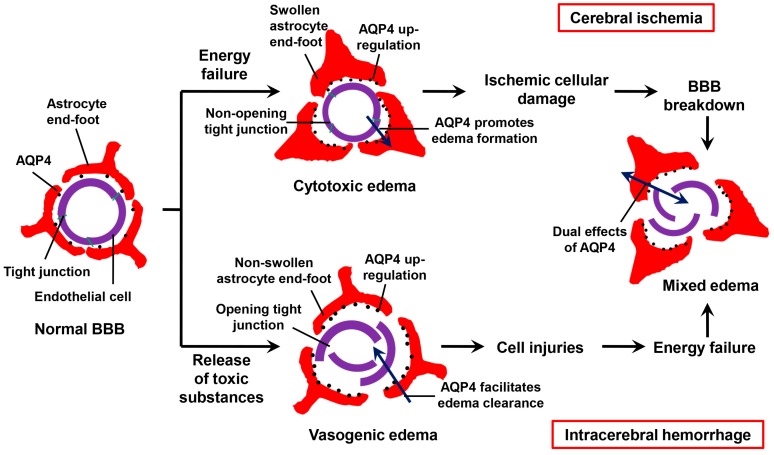
Mechanisms of edema formation and the effects of AQP4 in cerebral ischemia and intracerebral hemorrhage (ICH). In cerebral ischemia, AQP4 promotes water entry into perivascular astrocyte end-feet resulting in cytotoxic edema. As further ischemic cellular damage evolves, the mechanism shifts into vasogenic edema. During ICH, release of multiple toxic substancescauses disruption of blood–brain barrier (BBB), which gives rise to vasogenic edema. AQP4 facilitates the reabsorption of edema fluid from the extracellular space. Then secondary cellular injury leads to energy failure causing cytotoxic edema. Thus, mixed brain edema exists in the two types of stroke but the predominant type is different [17,58,69,70].

In the early stage of cerebral ischemia, the decline of cerebral blood flow causing hypoxia results in impairment of Na^+^/K^+^ ATPase. The energy failure leads to accumulation of intracellular sodium, which draws water into the cell inducing cytotoxic edema [58]. The development of ischemic cellular damage causes breakdown of BBB, giving rise to leakage of plasma proteins into extracellular space. The involved mechanisms are complex, including reverse pinocytosis, disputed Ca^2+^ signaling and action of other agents such as vascular endothelial growth factor (VEGF) and matrix metalloproteinases (MMPs) [17]. With the advance of BBB disruption, vasogenic edema occurs and even hemorrhagic conversion appears in some cases. The two types of cerebral edema coexist during the non-acute phase of cerebral ischemia [59] (Figure 2).

The effects of AQP4 on brain edema after cerebral ischemia are mainly investigated by AQP4 inhibition models, including AQP4 knockout, AQP4 depolarized distribution and AQP4 gene silencing. Because cytotoxic edema is the predominant type in the early stage, AQP4 inhibits formation of edema based on the knowledge mentioned above. The first report by Manley et al. showed brain edema was decreased in AQP4-deficient mice at 24 h after permanent MCAO [36]. Then several studies reveal similar results in different ischemic models. AQP4 small interfering RNA (siRNA) relieved cellular edema at 6 h after MCAO [60]. Also, it was shown that AQP4 deletion reduced brain edema at 24 h after transient MCAO [61]. Yang and colleagues demonstrated that AQP4 knockdown by siRNA led to reduced brain edema accompanied by a higher apparent diffusion coefficient (ADC) value from 0 h to 12 h after hypoxia–ischemia established by suturing the bilateral carotid arteries in newborn piglets [62]. Moreover, in global cerebral ischemia models, AQP4 knockout alleviated brain water content at 24 h as well as astrocyte swelling in brain slice [63]. Since AQP4 plays dual roles in the two types of edema and the brain edema of cerebral ischemia during non-acute phase is mixed one, the effects of AQP4 during the phase are complex. It was reported brain edema was reduced in α-syntrophin deficient mice at 48 h and 72 h after transient MCAO [64]. Meanwhile, brain water content was increased in wild type mice compared with AQP4 deletion mice at 3 days and 5 days after severe global cerebral ischemia produced by transient four-vessel occlusion [65]. However, a recent study showed AQP4 deletion increase brain edema determined by MRI especially at 3 days and 7 days after transient MCAO [66]. Thus, the roles of AQP4 on brain edema in non-acute cerebral ischemia are complex and possibly related to models and detecting methods. In astrocytes culture, AQP4 siRNA protects against water influx in the formation of astrocyte swelling, while delays water clearance in the resolution of astrocyte swelling after OGD/reoxygenation [67]. In addition, it was reported thrombin preconditioning up-regulated AQP4 with predominant AQP4-M1 isoform at 24 h after MCAO, leading to reduction of brain edema [68]. It was suggested the ratio of AQP4-M23 and AQP4-M1 may be crucial for edema formation and elimination.

### 3.4. Effects of AQP4 on Ischemic BBB and Neural Cells Injury

In addition to the influence of brain edema, AQP4 can elicit other biological effects as mentioned above. As a result, AQP4 may affect ischemic BBB and neural cells injury not only dependent on regulation of brain edema. Since AQP4 is highly concentrated in the important location of BBB, it is regarded to play important roles in maintaining BBB integrity in development and mature individuals [71,72]. However, in a global cerebral ischemic model, AQP4 deletion reduced BBB disruption measured by Evans blue dye extravasation [65]. Similar result was also found in a transient MCAO model [61]. It may result from inhibition of secondary injury to BBB by reduction of brain edema in AQP4-null mice, but dynamic and systematical observation to ischemic BBB injury remains to be carried out in the future. Moreover, it was demonstrated AQP4 knockout improved outcome and neurological function, reduced infarction volume, increased neuronal survival, and blocked apoptosis and inflammatory response after cerebral ischemia, which was consistent with brain edema reduction [61,62,63,65]. However, AQP4 deletion was reported to be beneficial at long term (14 days after MCAO) with neuronal survival improvement and neuroinflammation reduction without a direct effect on edema formation, suggesting a complex role of AQP4 in the ischemic pathophysiological cascades [66]. In an in vitro experiment, AQP4 siRNA also attenuated astrocytes injury induced by OGD/reoxygenation [52,73]. Nevertheless, one study revealed reverse results that AQP4 deletion aggravated inflammation and promoted neuronal loss at 24 and 72 h after MCAO [74]. Meanwhile, in chronic cerebral ischemia (35 days after MCAO), AQP4 knockout had more severe brain atrophy and more neuronal loss as well as impaired astrocyte proliferation and glial scar formation [75]. In summary, the effects of AQP4 on cerebral ischemia may be very complex and include several mechanisms. In the early stage, owing to the inhibition of relatively single cytotoxic edema, AQP4 should exhibit protective effects. As the disease development and long-term existence of mixed edema, effects of AQP4 are very complex, which are determined by the predominance of dual effects on the two types of edema and certain neuroprotective effects. As to chronic ischemia, AQP4 probably contributes to facilitative effects on neurorestoration because of its roles in astrocytes migration and neurogenesis promotion.

## 4. Intracerebral Hemorrhage (ICH)

### 4.1. Expression of AQP4

The research related to ICH is only limited to few animal model studies due to the absence of proper cell models. Animal models of ICH contain autologous blood injection, bacterial collagenase injection and spontaneous ICH models, and the former two are the most commonly used [76]. In collagenase models, bacterial collagenase disrupts the basal lamina of blood vessels and causes blood leakage leak into the surrounding tissue. Both have merits and demerits and they differ in ways that influence outcome. However, none of them mimic the pathophysiologic course of human spontaneous ICH, causing relative lag in experimental ICH research [77].

Several articles have revealed AQP4 expression is up-regulated from 3 h after ICH, reaches the peak at 2–5 day, and lasts for at least 14 days, which is not different between autologous blood [78,79,80,81,82,83] and collagenase models [84,85,86]. Meanwhile, it was also reported AQP4 polarity was disturbed in spite of AQP4 up-regulation [87]. Moreover, AQP4 is internalized following ICH and the lysosome is involved in degrading the internalized AQP4 [86]. No research has referred to AQP4 expression in ICH patients.

Certain signal transduction pathways are crucial for regulation of AQP4 following ICH. It was reported that nuclear factor κB (NF-κB) participated in AQP4 up-regulation [80]. Furthermore, Chu et al. demonstrated that activation of JNK and ERK pathways, ERK pathway, and JNK and p38-MAPK was responsible for increase of AQP4 by VEGF, granulocyte-colony stimulating factor (G-CSF) and erythropoietin (EPO), respectively [88,89,90]. This indicates MAPK pathways also play important roles in AQP4 up-regulation.

### 4.2. Effects of AQP4 on Hemorrhagic Edema

Brain edema following ICH remains complicated. Studies using animal models revealed autologous blood injected into the brain causes the activation of thrombin, plasminogen activator and urokinase. These substances activate inflammatory cells and disrupt BBB, leading to vasogenic edema. The above mechanism starts at several hours and peaks at several days after ICH. Subsequently, secondary cellular injury due to the substances from CNS cells disruption and red blood cells lysis leads to cytotoxic edema. Thus, these degradation products maintain mixed edema, which lasts about two to three weeks. As a result, ICH results in multiple forms of edema while the predominant type is probably vasogenic [69,70] (Figure 2). In general, brain edema and BBB disruption following ICH are more severe than cerebral ischemia, which usually greatly contribute to ICH-induced neurological deficits.

Several studies have showed that the changing trend of AQP4 is parallel with brain edema after ICH [82,83]. However, the effects of AQP4 cannot be determined without further investigation. Tang and colleagues first observed the roles of AQP4 in ICH using AQP4 knockout mice. They focused on brain edema surrounding hematoma and used two methods to measure brain water content and brain specific gravity. The advantage of the latter is that it is possible to obtain reliable results in tissue samples as small as 10–30 mg. Both methods showed AQP4 deletion aggravated brain edema at 1, 3 and 7 days after ICH [78]. Similar results were obtained by Chu et al. [88,89]. Considering AQP4 contributes to clearance of vasogenic edema and this type is predominant in ICH, it is probable that AQP4 mainly acts on elimination of hemorrhagic edema. In addition, brain edema following ICH also has close relation with disruption of AQP4 polarized distribution [87]. As to patient studies, one study on polymorphism in AQP4 genes suggests AQP4 gene variant, single nucleotide polymorphism (SNP) rs1054827, is independently associated with brain edema after ICH [91].

### 4.3. Effects of AQP4 on Hemorrhagic BBB and Neural Cells Injury

Disruption of BBB is an important pathophysiological change after ICH and contributes to formation of vasogenic brain edema, giving rise to poor prognosis. It was found BBB function measured by Evans blue extravasation was worsened by AQP4 deletion at 1, 3 and 7 days after ICH compared with wild type mice [78,88,89]. As for the morphology of BBB, electron micrographs showed AQP4 deletion resulted in swelling and irregular capillary endothelial cells with opening of tight junction [78,90]. Meanwhile, AQP4 knockout reduced expression of tight junction proteins including occludin, zonula occluden-1 (ZO-1) and claudin-5 [90]. These results suggest AQP4 may have protective effects on BBB disruption after ICH both morphologically and functionally.

The presence of AQP4 gene also improves neurological function, increases the survival rate and inhibits neuronal death and apoptosis after ICH [78,88,89,92]. Chu et al. first investigated the mechanisms involved in AQP4’s effects on apoptosis. In this work, AQP4 deletion increased apoptosis and the cell types involved were predominantly neurons and astrocytes. The apoptosis-related proteins including activated caspase-3 and caspase-8 were increased. Meanwhile, higher levels of tumor necrosis factor-α (TNF-α) and interleukin-1β (IL-1β) as well as their receptors were detected in AQP4 knockout mice. The inhibitors of the two cytokines alleviated cells apoptosis after ICH. It suggests AQP4 deletion increases apoptosis following ICH, and the underlying mechanism may be that cytokines, especially TNF-α and IL-1 β, initiate the apoptotic cascade and activates caspase-3 and caspase-8 [92]. Therefore, AQP4 may affect ICH and even other CNS diseases by edema independent of neuroinflammatory pathways.

Furthermore, AQP4 can be located in downstream of certain drugs and proteins, thus mediates their effects on ICH. Although several articles reported some factors reduced brain edema and BBB disruption with a decrease of AQP4 [81,85], it is still hard to conclude the effects are associated with AQP4. Chu and colleagues tested the effects of VEGF, G-CSF and EPO on brain edema, BBB permeability and cells injury following ICH and examined whether they were AQP4 dependent using AQP4 deletion mice. They found these effects were associated with AQP4 [88,89,90]. Thissuggests AQP4 can mediate other neuroprotective factors’ effects as downstream pathways.

## 5. Subarachnoid Hemorrhage (SAH)

SAH is a devastating subtype of stroke with high mortality, which is mostly followed by aneurysm rupture [93]. Early and delayed brain injuries are included in the pathophysiology. Early brain injury occurs immediately after SAH and lasts up to 72 h. Brain edema appears at early phase of SAH due to disruption of BBB via multiple mechanisms causing vasogenic edema, which is probably the major type [94]. Meanwhile, cytotoxic edema is detected also at early stage owing to ischemic insult [95]. Delayed brain injury mainly results in vasospasm and brain edema in this stage is similar to cerebral ischemia.

Research on SAH patients shows AQP4 is up-regulated on the astrocytic processes with loss of polarization [96]. Till now, studies in SAH models mainly focus on effects of AQP4 on early brain injury. AQP4 is increased at 6 h after SAH and maintains high levels within 72 h [97,98,99,100,101]. Moreover, one study indicates an increase of AQP4 at 7 days after SAH, which is located in the phase of delayed brain injury [102]. Thus, AQP4 may be up-regulated early after SAH and last for a relatively long time.

AQP4 may play a dual role in brain edema after SAH due to the mixed type of edema. Tait et al. demonstrated AQP4 knockout markedly reduced brain edema and BBB disruption at 6 h and 24 h after SAH with an increase of intracranial pressure and aggravation of neurological function [98]. In addition, it was shown that there was no improvement in neurological deficits and neuroinflammation at 7 days after SAH in AQP4 deletion mice compared with wide type control mice [103]. Therefore, AQP4 may play opposite roles in the early and delayed brain injury and further research using AQP4-null mice is urgently required.

## 6. Conclusions

As the most abundant AQP in the CNS, the expression of AQP4 is increased in three kinds of cerebrovascular diseases, including cerebral ischemia, ICH and SAH. The direct effect on the related brain edema is the basis of the action of AQP4 for these diseases. However, vasogenic edema and cytotoxic edema may simultaneously appear in these diseases and the roles of AQP4 in the two types of edema are opposite. Meanwhile, AQP4 also shows other biological effects in addition to the effect on brain edema. Thus, the roles of AQP4 in these diseases are relatively complicated, which may be determined by the balance between its effect on the predominant brain edema type and other biological effects. Furthermore, it may also play different roles in alternative phase of the diseases. Thus, the effects of AQP4 on cerebrovascular diseases remain to be investigated, which may become the theoretical basis of AQP4 regulation treatment.

AQP4 plays an important role in the formation and clearance of brain edema, and appropriate regulation of AQP4 may treat brain edema from perspectives of mechanism as a remedy of the disadvantages of the current common treatment, such as dehydrant or surgery. Although AQP4 gene knockout is an excellent tool to study AQP4, it cannot be used clinically. Furthermore, AQP4 gene silencing or over-expression is only locally applied, and invasive procedures are required. Therefore, the highly selective antagonist or agonist of AQP4 may be a better choice. It is well-known that neuromyelitisoptica (NMO) is characterized by autoantibodies directed against AQP4. However, purified NMO-IgG injected intravenously increased brain edema and infarct size at 24 h after MCAO [103]. Moreover, Igarashi et al. developed an AQP4 inhibitor TGN-020 and found intraperitoneal injection of TGN-020 reduced brain edema and infarct size at 24 h after MCAO. Nevertheless, no other research has duplicated their results [104]. Thus, a highly selective antagonist or agonist of AQP4 that can be used systemically remains to be further developed, and it promises to become a novel, effective measure of treating cerebrovascular diseases.
